# STI Knowledge in Berlin Adolescents

**DOI:** 10.3390/ijerph15010110

**Published:** 2018-01-10

**Authors:** Frederik Tilmann von Rosen, Antonella Juline von Rosen, Falk Müller-Riemenschneider, Inken Damberg, Peter Tinnemann

**Affiliations:** 1 Charité—Universitätsmedizin Berlin, Institute for Social Medicine, Epidemiology and Health Economics, 10117 Berlin, Germany; an_pa@uni-bremen.de (A.J.v.R.); falk.mueller-riemenschneider@nuhs.edu.sg (F.M.-R.); peter.tinnemann@charite.de (P.T.); 2 Institute for Public Health and Nursing Research, University of Bremen, 28359 Bremen, Germany; idamberg@uni-bremen.de; 3 Saw Swee Hock School of Public Health, National University of Singapore, Singapore 117549, Singapore; 4 Akademie für Öffentliches Gesundheitswesen, 40472 Düsseldorf, Germany

**Keywords:** sexual health, sexually transmitted diseases, sexually transmitted infections, adolescent health, Berlin, Germany

## Abstract

Sexually transmitted infections (STIs) pose a significant threat to individual and public health. They disproportionately affect adolescents and young adults. In a cross-sectional study, we assessed self-rated and factual STI knowledge in a sample of 9th graders in 13 secondary schools in Berlin, Germany. Differences by age, gender, migrant background, and school type were quantified using bivariate and multivariable analyses. A total of 1177 students in 61 classes participated. The mean age was 14.6 (SD = 0.7), 47.5% were female, and 52.9% had at least one immigrant parent. Knowledge of human immunodeficiency virus (HIV) was widespread, but other STIs were less known. For example, 46.2% had never heard of chlamydia, 10.8% knew of the HPV vaccination, and only 2.2% were aware that no cure exists for HPV infection. While boys were more likely to describe their knowledge as good, there was no general gender superiority in factual knowledge. Children of immigrants and students in the least academic schools had lower knowledge overall. Our results show that despite their particular risk to contract an STI, adolescents suffer from suboptimal levels of knowledge on STIs beyond HIV. Urgent efforts needed to improve adolescent STI knowledge in order to improve the uptake of primary and secondary prevention.

## 1. Introduction

Sexually transmitted infections (STIs) are a serious public health problem worldwide, with an estimated one million new infections each day [1]. They have a wide range of negative consequences on individual health, ranging from physical discomfort to infertility, malignancy, severe maternal and foetal pregnancy complications, and loss of life [2]. Beyond the detrimental effect on individual health, STIs also represent a significant economic burden. It is estimated that more than ten billion dollars per year are spent on STIs other than human immunodeficiency virus (HIV) in the United States alone [3]. The most frequent viral STI pathogens are human papillomavirus (HPV), herpes simplex virus 2 (HSV-2), HIV, and Hepatitis B. The most widespread bacterial STIs are chlamydia trachomatis, gonorrhoea, and syphilis [4,5].

Although HIV prevalence in Germany is still low compared to most other European countries [6], there has been a marked rise in the number of newly diagnosed cases of HIV resulting from both homosexual and heterosexual intercourse, as well as intravenous drug use over the last five years [7]. An even higher increase in incidence has been observed in other STIs: chlamydia, commonly regarded as the most frequent cause of female infertility in developed countries [8,9], is becoming widespread in Germany [10,11]. The number of newly diagnosed syphilis cases in Germany has risen more than fourfold since 2000 [12,13], and gonorrhoea cases are becoming more widespread, with increasing antibiotic resistance being an additional problem [14,15]. Furthermore, HPV-related neoplasia is a growing problem. For example, vulvar cancer, often linked to an infection with HPV, has greatly increased in Germany over the last decade [16]. HPV is also frequently transmitted in oral and anal sexual practice, causing high rates of local infection of the oropharyngeal and anal regions [17,18]. This is likely to be the cause of the increasing rates of HPV-related neoplasia of the head and neck [19], and of the anus [20]. 

Despite increasing numbers of new infections with STIs, the limited existing research indicates a considerable ignorance regarding the existence and dangers of STIs other than HIV in Germany. One study found that in a representative sample of adults across Germany, a majority had never heard of syphilis, gonorrhoea, hepatitis, genital herpes, chlamydia, and HPV. Only 6% were aware of HPV and 14% of chlamydia as the two most frequent viral and bacterial STIs [21]. Another study amongst adolescents in two cities in Northern Germany discovered that participants perceive the likelihood of infection with HIV much higher than that of the much more prevalent infection with HPV [22]. Furthermore, adolescents were found to be largely ignorant of the existence of chlamydia, even in an urban setting of particularly high prevalence [23]. 

One reason for this large-scale ignorance might be that STIs other than HIV escaped the attention of German public health authorities and sexual health educators for a long time. For example, over the last two decades, the Federal Centre for Health Education (Bundeszentrale für gesundheitliche Aufklärung, BZgA) as the national authority for health education and health promotion has launched a multitude of campaigns warning of the risk of HIV, but an awareness campaign for other STIs, such as hepatitis B or chlamydia, was only launched in 2016 [24].

Due to more frequently changing sexual partners and lower rates of correct and consistent contraceptive use, adolescents and young adults are particularly at risk of contracting and transmitting STIs [25,26,27]. For women it has been argued that, the risk of infection in adolescence or early adulthood is elevated further due to a greater anatomical susceptibility to certain STIs at young age [28,29]. Indeed, studies show a higher incidence of STIs in this age bracket [30,31,32]. One review estimated that adolescents and young adults account for 50% of new infections with STIs, while representing only 25% of the sexually active population [33]. Young people are also likely to suffer most from long-term sequelae, for example if an infection with an oncogenic type of HPV leads to neoplasia years or decades later [34], or if they experience hypofertility at a later stage caused by untreated infection with chlamydia [35]. Therefore, adolescents can be regarded as a particularly suitable audience for primary and secondary STI prevention. 

Condom use is an effective method to prevent infection with an STI [36,37]. Moreover, several STIs can be readily prevented through vaccination (Hepatitis B, HPV) [38,39]. Others, such as chlamydia, gonorrhoea, or syphilis, are curable with appropriate antibiotic regimens, thus enabling the prevention of long-term sequelae [40,41,42]. However, awareness of the existence and risk of different STIs are prerequisites to actual utilization of preventive and curative options. 

Currently, readily available prevention methods are not widely utilized in Germany. One study found that only 39% of boys and 31% of girls used a condom in their last sexual contact [43]. Only 11.3% of women under 25 participated in the recommended chlamydia screening in 2015 [24], and less than a third of girls are vaccinated against HPV at the average age of sexual debut [44]. This is despite the fact that both adolescent HPV vaccination and chlamydia screening are strongly endorsed by German medical bodies and are fully covered by statutory health insurance [45,46]. 

Considering the limited existing research on adolescent STI knowledge in Germany, we assessed self-reported and factual knowledge on different STIs amongst 9th-graders in Berlin, Germany. Specifically, we assessed the extent to which participants were aware of different STIs and how they self-rated their knowledge. Factual knowledge on STI curability and on the existence of vaccines for STIs was tested. Results could help schools, parents, and other sexual health educators to address particular “areas of need”, in order to raise awareness and knowledge amongst a population disproportionately at risk of acquiring STIs.

## 2. Methods

### 2.1. Study Design 

Data on STI awareness was collected within the framework of a larger survey on the knowledge of sexual health issues amongst Berlin adolescents. Details of the study and its population and methodology have been partially described elsewhere. It was demonstrated that adolescents were ill-informed on the important issue of emergency contraception [47]. Furthermore, adolescents’ preferences regarding online sexual health resources were assessed [48]. The survey was conducted throughout the year 2012 in grade nine of secondary schools in Berlin.

The study was conducted with the approval of the Ethics Committee of the Charité-Universitätsmedizin Berlin and the Berlin Senate’s Department for Education, Youth and Science. In accordance with Berlin state law, written parental consent was mandatory for all students who had not yet reached 14 years of age, and a favourable vote of the parent-teacher-conference was a prerequisite for a school’s participation. 

### 2.2. Sampling and Data Collection

All public secondary schools in Berlin were contacted by telephone and email with in-depth information on the study and a request to include the school in the sample. Schools allocated regular lessons in which the study was conducted using paper questionnaires. Students were informed of the aim of the study and the range of topics addressed. It was pointed out that participation was voluntary and anonymous, and that the survey did not represent a formal school assignment or otherwise affected school grades. Following the collection of questionnaires, students were provided with the correct answers to all knowledge questions.

### 2.3. Questionnaire

The questionnaire was designed by the authors to assess self-evaluated and actual knowledge on different STIs, amongst other sexual health questions. A pre-test in one school class was conducted and the comments led to minor modifications in the wording of questions.

In the general part of the questionnaire, students were requested to state their age and gender. Furthermore, students were asked to provide their parents’ place of birth to assess migratory background. To safeguard parental informational self-determination in accordance with Berlin Senate policy, options were limited to “Germany” and “abroad”, and no information on the time of migration was collected.

Furthermore, the variable of school type was coded for each participant to assess the differences between the three types of Berlin secondary schools: the most academically selective type of University-Preparatory Schools (Gymnasium), Comprehensive Secondary Schools (Integrierte Gesamtschule) with the option to qualify for university access, and the least academic school type—Comprehensive Secondary Schools without this option. For clarity, these three school types were reported below as “highest academic tier”, “intermediate academic tier”, and “lowest academic tier”.

In the part on STIs, students were first asked to self-evaluate their knowledge of the seven most frequent bacterial and viral STIs: HIV/AIDS, syphilis, genital herpes, hepatitis B, gonorrhoea, chlamydia, and HPV. A Likert-Scale was employed, with the options to rate knowledge as “good”, “rather good”, “mediocre”, “rather bad”, and “bad”, and a further option to select “I have never heard of this STI”. Furthermore, factual knowledge was tested by asking students to state whether a reliable cure and/or a vaccination exists for the following STIs (correct responses in brackets, according to the current Centers for Disease Control and Prevention (CDC) Guidelines [49]): HIV (no cure, no vaccination), hepatitis B (no cure, vaccination exists), chlamydia (curable, no vaccination), HPV (no cure, vaccination exists), and genital herpes (no cure, no vaccination). 

### 2.4. Statistical Analysis

IBM SPSS Statistics Version 25 (SPSS Inc., Chicago, IL, USA) was employed for data analysis. Frequencies were computed for all items. We used chi-square statistics to test for bivariate relationships between the independent variables gender, migratory background, and school type, and the outcomes of self-reported awareness and knowledge in the factual questions on STIs.

Using multiple regression models, we quantified the effect of demographic variables on outcomes. Since age, gender, migratory background, and academic standing had all been shown to be predictors of sexual health knowledge in different previous studies [50,51,52], they were maintained as factors in all of the analyses. To account for the possible effect of clustering by school or class, a mixed multilevel regression model (SPSS GENLINMIXED) was employed and school and class included as random effects. Odds ratios and confidence intervals were calculated from regression. For clarity of results, outcome categories were dichotomized for regression. The regression outcome variables were thus “high knowledge” (response either “good” or “rather good” knowledge) on individual STIs, “never heard” for individual STIs, and “correct response” for each of the knowledge questions. Robust estimation was used to take into account possible violations of model assumptions. Missing cases were excluded from statistical analyses. 

The Strengthening the Reporting of Observational Studies in Epidemiology (STROBE) statement [53] was followed in the presentation of the methods and results of this study. The statistical methodology used was approved by the Competence Center for Clinical Trials of the University of Bremen. 

The original dataset of the study cannot be made available to the general public due to the constraints placed on data availability by the Berlin Senate’s Department for Education, Youth and Science. The dataset can be obtained upon reasonable request from the corresponding author.

## 3. Results

### 3.1. Study Participants

We successfully contacted the heads of the biology departments in 142 out of 287 schools. Subsequently, 13 schools with a total of 61 ninth grade classes agreed to participate. The limited time to teach the demanding state curriculum was most frequently given as the reason for non-participation. 

Participating schools hailed from seven of the twelve Berlin City Districts (Mitte, Pankow, Charlottenburg-Wilmersdorf, Spandau, Steglitz-Zehlendorf, Treptow-Köpenick, and Marzahn-Hellersdorf) and included very diverse settings. Schools in former East and West Berlin were included, as were schools from inner-city to suburban, and from the most wealthy to relatively impoverished city areas [54].

During the lessons that were allocated by participating schools, 1190 students were in attendance. Ten students aged 13 failed to provide parental consent and could thus not participate. Two students elected not to take part, and one very recently arrived immigrant was unable to read German. Thus, a total of 1177 students participated.

Of participants providing gender information, 547 were female (46.5%) and 605 (51.4%) were male. Age ranged from 13 to 16 years, with a mean age of participants of 14.6 (SD 0.8). For migratory background, 544 (46.2%) had two German-born parents, 260 (22.5%) participants reported one, and 352 (30.4%) two parents of foreign birth. Participants were virtually equally spread across the three school types, with 390 (33.1%) participants attending a school of the lowest, 395 (33.6%) of the intermediate, and 392 (33.3%) of the highest academic tier. 

### 3.2. Self-Reported STI Knowledge

Self-evaluated knowledge regarding the most widespread bacterial and viral STIs, sorted in order of decreasing awareness is shown in Table 1. This order will be followed in subsequent tables. HIV was known to virtually all participants, with many stating good or rather good knowledge. Knowledge and awareness were visibly lower for other STIs, of which the most frequently known infection was hepatitis B. Despite being the bacterial STI with the highest prevalence, chlamydia was the infection with the lowest proportion of participants claiming good knowledge and the lowest rate of awareness.

Distribution of self-evaluated knowledge by gender is shown in Table 2. Association between gender and reported knowledge was statistically significant for all STIs, except for hepatitis B and chlamydia. Female respondents reported lower knowledge and were more likely to state complete lack of awareness for each of the STIs apart from chlamydia. While chlamydia was the STI with the lowest awareness overall and amongst male participants, gonorrhoea was the infection least known to girls in the sample.

A table of differences by gender, migrant status and school type of participants who selected the “never heard of” option for the presented STIs can be found in Supplementary File 1. Students from the intermediate tier of schools were generally least likely to have never heard of the different STIs. Students of two foreign-born parents were most likely to be fully unaware of the existence of all STIs, bar hepatitis B. 

### 3.3. Factors Associated with Self-Reported STI Knowledge in Multivariable Analysis

Table 3 depicts the results of the regression model for high self-evaluated knowledge. For all STIs bar hepatitis B and chlamydia, female respondents were significantly less likely to evaluate their knowledge as “good” or “rather good”. 

The results of the regression model for the outcome of “unawareness” of the different STIs are presented in Table 4. While there were no significant differences for HIV, hepatitis B, and chlamydia, female students were significantly more likely to have never heard of HPV, syphilis, and gonorrhoea. Students with an immigrant background were more likely to have never heard of genital herpes and syphilis. 

### 3.4. Factual STI-Knowledge

#### 3.4.1. Bivariate Analyses

Correct response rates to the knowledge questions on the curability and existence of vaccines for STIs are presented in Table 5. While it was widely known that no reliable cure has been found for HIV to date, knowledge was much lower regarding the curability of other STIs. For vaccination, again a majority knew that no vaccination protects from HIV, and slightly less than half knew that a vaccination exists for hepatitis B. Knowledge was low for the other STIs, and only 10.8% of participants were aware of the existence of an HPV vaccine.

Results by gender varied depending on the STI and there was no trend suggesting general knowledge superiority of either gender. While awareness of the HPV vaccine was low overall, it was significantly higher among boys than girls. 

Students with migratory background overall tended to have lower rates of correct responses. For example, of students with two German-born parents, 370 out of 528 (70.1%) were aware that no vaccination was available for HIV and 463 out of 529 (87.5%) knew that HIV could not reliably be cured. Corresponding numbers with both parents born abroad were 177/343 (51.6%) for vaccination and 252/339 (74.3%) for curability (*p* (from χ^2^) <0.001 and <0.001). While association was not significant for most STIs, lower proportions of children of immigrants selected the correct response on all of the questions but on curative options for HPV and genital herpes. A table with STI knowledge by migratory background can be found in Supplementary File 2.

For all questions bar on the curability of infection with HPV, it was students from the intermediate academic tier of schools who had the highest rate of correct responses. There was no clear knowledge difference between students from the lowest or the highest tier of schools. The full results for knowledge differences by school type can be found in Supplementary File 3.

#### 3.4.2. Multivariable Analyses

Multivariate analysis was performed for the questions on curability and vaccination options for HIV, Hepatitis B, HPV, genital herpes, and chlamydia. The dichotomous outcome categories were “correct response” vs. “other response”. Results are presented in Table 6.

Table 7 shows the results from the multivariable regression model for the outcome variable of correct responses on cure and vaccination questions. As in bivariate analyses, autochthonous German students were more likely to know the lack of curative and vaccination options for HIV, as were students from the intermediate tier of schools. Male students were again significantly more likely to know that a vaccination exists for HPV. 

## 4. Discussion

We measured self-rated and factual knowledge regarding different STIs in the framework of a cross-sectional study assessing the sexual health knowledge of Berlin adolescents. We encountered a high participation rate (1177/1179) despite the explicit emphasis on the voluntary nature of participation. It was assessed whether students had heard of different STIs, how they would describe their own knowledge, and whether they were able to state which STIs were curable and for which a vaccination exists. 

With a mean participant age of 14.6 years, population-level data suggests that a majority of adolescents in our study had already experienced some form of sexual contact or intimacy, although most have not engaged in penetrative sexual intercourse [43]. Across Germany, the mean age of sexual debut is 14.9 years for female and 15.1 years for male adolescents [55].

As expected, nearly all of the students had heard of HIV, and a majority rated their knowledge as (rather) good and knew that HIV could neither be cured nor vaccinated against. However, overall knowledge for other STIs was much less satisfactory, with low self-reported knowledge and high levels of ignorance regarding individual STIs. For example, more than 46% of participants had never heard of chlamydia and merely 18% knew that chlamydia can be cured. This widespread lack of knowledge is in line with previous studies in Germany on both adolescents and the population at large [21,22]. Results from adolescents in South-East England point towards a similar level of ignorance [56], whereas Swedish studies show between 86% [57] and 96% [58] of adolescents to be aware of chlamydia. This lack of awareness that is shown by our study is particularly noteworthy given the fact that chlamydia is the most frequent bacterial STI [4], has a particularly high incidence among adolescents and young adults [59,60], and is a frequent reason for infertility later in life [9]. Despite the ready and cost-free availability of chlamydia screening and treatment to German adolescents, our results show the target population is hardly aware of the disease’s existence.

Another STI for which there was a widespread lack of knowledge was HPV. Despite being the STI with the highest prevalence [4], more than a third of students responded to have never heard of it, and fewer than 13% described high knowledge. Results for the factual questions were dire, with less than 2.2% of respondents knowing that there is currently no treatment to cure HPV infection, and that the HPV vaccine is only known to 10.8% of respondents. This is visibly lower than the rates that are found amongst adolescents in a previous study in Germany [51] and in other European countries [61,62,63]. In contrast to these previous studies, the male participants in our sample were significantly more likely to be aware of the HPV vaccine, shown both in bivariate and regression analysis. This is especially surprising given that the HPV vaccine is primarily marketed to a female audience in Germany: in all German states except for Saxony, HPV vaccination is exclusively recommended for female adolescents [64], and only few statutory health insurance providers cover male HPV vaccination [65].

Condoms can prevent infection with STIs. However, their use requires motivation. Research shows that adolescents regard condoms primarily as a method to prevent pregnancy. If STI prevention is considered at all, it is mainly the risk of HIV that is taken into account [56,66]. However, most sexually active adolescents in Germany rely on hormonal methods of contraception to prevent pregnancy, with only a minority employing condoms instead or concurrently [43]. HIV, due to its low prevalence in Germany, is unlikely to be an effective motivator for condom use, at least in heterosexual intercourse. Our results show, that other STIs—most of which are much more prevalent than HIV—are relatively little known to adolescents. This lack of knowledge is likely to diminish motivation to use condoms and/or access other methods of STI prevention, both primary (such as HPV or hepatitis B vaccination) and secondary (such as Pap tests or chlamydia screening). If adolescents and young adults are to make a truly informed choice on the uptake of preventive options, the level of STI awareness and knowledge needs to be improved. 

In our results, for most STIs, even groups that are usually shown to have superior sexual health proficiency (such as female adolescents, the academically-advantaged, and non-ethnic minority students [43,51,67,68]) have only non-satisfactory knowledge. We regard this as indicative that schools, parents, primary care physicians, public health authorities, and other providers of sexual health information fail to communicate the most basic facts about STIs beyond HIV, even to the most accessible population groups. The recent launch of the first (non-HIV) STI awareness campaign by the BZgA is a much needed initiative towards improving STI knowledge. However, in a country with compulsory school education, we regard schools as the most promising vector to multiply relevant health information and reach virtually all adolescents. For this, it is imperative that STIs and STI prevention are explicitly included in the school curriculum. The current state curriculum in Berlin, unfortunately, falls short in this respect Condoms are the only prevention method, and “HIV/AIDS” the sole STI specifically included in the curriculum [69]. Comprehensive teacher-delivered school tuition on STIs and preventive instruments could address the widespread information gap that is highlighted by our study, as could the co-optation of external providers of STI prevention education into schools [70]. Beyond schools, STI information can—and should—also be spread through other means, for example through healthcare professionals [71], social media channels [72], mass media campaigns [73], and through peer education programmes [74]. A multidimensional approach combining different channels of access to adolescents bears the promise to improve STI knowledge, and thus potentially curtail the increasing rates of new infections and lower the rate of long-term sequelae of infection.

### Strengths and Limitations

The relatively large number of participants from a sample of schools of all three types of Berlin public schools represents a strength of the study, as does the virtually complete rate of participation.

Only 13 out of a total of 287 eligible schools, however, took part in the study. Despite schools hailing from very diverse social and geographical settings, there might be a systemic difference between participating and non-participating schools, for example in terms of the teaching bodies’ interest and openness regarding sexual health education. 

Furthermore, students were asked to evaluate their knowledge regarding STIs on a multiple choice questionnaire, which might have led to the over-reporting of knowledge as compared to a questionnaire in which participants were to list STIs in an open question. 

## 5. Conclusions

Sexually transmitted infections pose a serious threat to individual as well as public health. Despite different effective instruments of primary and secondary prevention being readily available, their uptake is impeded by widespread lack of knowledge. This is especially true for adolescents and young adults, an age group that is particularly at risk of contracting an STI. Our study shows that across demographic divides, adolescents in Berlin, Germany suffer from a low level of knowledge on all of the most frequent STIs apart from HIV, despite their growing incidence. It is crucial that this lack of knowledge is addressed and that adolescents are educated on the threats that are posed by different infections, and on the existing and readily available methods to prevent, detect, and cure STIs. Knowledge is a necessary prerequisite to making an informed choice regarding STI prevention, screening, and treatment and schools as well as health information providers need to address current knowledge deficits.

## Figures and Tables

**Table 1:** Self-rated knowledge of sexually transmitted infections (STIs) in order of decreasing awareness.

	Self-Rated Knowledge					
STI	Good n (%)	Rather Good n (%)	Mediocre n (%)	Rather Bad n (%)	Bad n (%)	Never Heard n (%)
HIV n = 1148 *	438 (38.2%)	379 (33.0%)	217 (18.9%)	58 (5.1%)	39 (3.4%)	17 (1.5%)
Hepatitis B n = 1136 *	142 (12.5%)	170 (15.0%)	292 (25.7%)	231 (20.3%)	150 (13.2%)	151 (13.3%)
Genital herpes n = 1130 *	109 (9.7%)	129 (11.4%)	264 (23.4%)	219 (19.4%)	143 (12.7%)	266 (23.5%)
Syphilis n = 1131 *	80 (7.1%)	129 (11.4%)	238 (21.0%)	188 (16.6%)	116 (10.3%)	380 (33.6%)
HPV n = 1129 *	66 (5.9%)	82 (7.3%)	163 (14.4%)	218 (19.3%)	178 (15.8%)	422 (37.4%)
Gonorrhoea n = 1125 *	84 (7.5%)	76 (6.8%)	170 (15.1%)	173 (15.4%)	141 (12.5%)	481 (42.8%)
Chlamydia n = 1134 *	63 (5.6%)	71 (6.3%)	154 (13.6%)	174 (15.3%)	148 (13.1%)	6.2%)

* number of responses included.

**Table 2:** Self-rated knowledge by gender.

		Self-Rated Knowledge						
STI	Gender	Good	Rather Good	Mediocre	Rather Bad	Bad	Never Heard	*p* (from χ^2^)
HIV	female	32.0%	35.1%	22.6%	5.5%	3.9%	0.9%	0.001
n = 1134 *	male	43.3%	31.0%	15.9%	4.7%	3.0%	2.0%	
Hepatitis B	female	11.3%	15.6%	25.6%	21.2%	13.2%	13.2%	0.96
n = 1122 *	male	12.9%	14.6%	25.9%	20.0%	13.2%	13.4%	
Genital herpes	female	6.8%	9.7%	23.7%	20.6%	13.1%	26.1%	0.03
n = 1116 *	male	11.4%	13.1%	23.1%	18.5%	12.2%	21.6%	
Syphilis	female	5.1%	8.1%	19.4%	16.0%	11.1%	40.3%	<0.001
n = 1117 *	male	8.2%	14.3%	22.7%	17.4%	9.2%	28.2%	
HPV	female	3.8%	5.3%	14.0%	19.8%	15.5%	41.6%	0.01
n = 1116 *	male	7.2%	9.2%	14.8%	19.1%	15.8%	33.9%	
Gonorrhoea	female	4.2%	6.2%	12.5%	15.7%	13.4%	48.0%	<0.001
n = 1112 *	male	9.6%	7.4%	17.5%	15.4%	11.7%	38.4%	
Chlamydia	female	5.8%	6.2%	11.6%	17.1%	13.1%	46.2%	0.45
n = 1121 *	male	4.8%	6.5%	15.1%	14.1%	12.9%	46.6%	

* number of responses included.

**Table 3:** Factors associated with high self-evaluated knowledge in multivariable analysis.

**Variable**	**HIV** n = 1127 **	**Hepatitis B** n = 1115 **	**Genital Herpes** n = 1109 **	**Syphilis** n = 1110 **
	**OR (95% CI)**	**OR (95% CI)**	**OR (95% CI)**	**OR (95% CI)**
Age (per year increase)	1.00 (0.83–1.19)	1.12 (0.89–1.41)	1.23 (0.92–1.66)	1.20 (0.83–1.74)
Female Gender *	0.71 (0.52–0.97) ***	1.03 (0.73–1.45)	0.65 (0.51–0.82) ***	0.55 (0.4–0.75) ***
Migratory Background *	0.58 (0.45–0.74) ***	0.96 (0.75–1.23)	1.04 (0.71–1.51)	0.69 (0.49–0.97) ***
Intermediate School Tier *	1.51 (0.72–3.17)	0.92 (0.76–1.10)	1.62 (0.59–4.43)	1.30 (0.66–2.55)
Highest School Tier *	1.13 (0.51–2.50)	0.66 (0.44–0.98) ***	0.66 (0.25–1.74)	1.19 (0.59–2.40)
	**HPV** n = 1109 **	**Gonorrhoea** n = 1106 **	**Chlamydia** n = 1114 **	
	**OR (95% CI)**	**OR (95% CI)**	**OR (95% CI)**	
Age (per year increase)	1.20 (0.87–1.65)	1.41 (1.07–1.86) ***	0.97 (0.72–1.32)	
Female Gender *	0.56 (0.40–0.78) ***	0.65 (0.43–0.99) ***	1.15 (0.69–1.91)	
Migratory Background *	1.05 (0.78–1.43)	0.77 (0.5–1.21)	0.89 (0.57–1.39)	
Intermediate School Tier *	1.12 (0.54–2.33)	0.69 (0.33–1.43)	1.09 (0.60–1.99)	
Highest School Tier *	0.44 (0.22–0.88) ***	0.58 (0.28–1.21)	0.44 (0.23–0.82) ***	

OR: Odds ratio; CI: Confidence interval; * Reference categories are male gender, no migrant background, and lowest school tier. ** number of participants included in the regression model; *** *p* < 0.05.

**Table 4:** Factors associated with unawareness of STIs in multivariable analysis.

**Variable**	**HIV** n = 1127 **	**Hepatitis B** n = 1115 **	**Genital Herpes** n = 1109 **	**Syphilis** n = 1110 **
	**OR (95% CI)**	**OR (95% CI)**	**OR (95% CI)**	**OR (95% CI)**
Age (per year increase)	0.96 (0.70–1.32)	0.92 (0.66–1.28)	0.86 (0.67–1.11)	0.87 (0.68–1.11)
Female Gender *	0.88 (0.65–1.19)	1.00 (0.61–1.65)	1.27 (0.85–1.92)	1.74 (1.37–2.22) ***
Migratory Background *	1.34 (0.96–1.88)	1.48 (0.87–2.51)	1.95 (1.52–2.49) ***	1.70 (1.08–2.67) ***
Intermediate School Tier *	0.61 (0.34–1.08)	0.81 (0.30–2.19)	0.47 (0.19–1.19)	0.63 (0.39–1.01)
Highest School Tier *	0.66 (0.36–1.24)	1.08 (0.48–2.42)	0.97 (0.42–2.24)	0.91 (0.52–1.56)
	**HPV** n = 1109 **	**Gonorrhoea** n = 1106 **	**Chlamydia** n = 1114 **	
	**OR (95% CI)**	**OR (95% CI)**	**OR (95% CI)**	
Age (per year increase)	0.95 (0.78–1.16)	0.77 (0.60–0.997) ***	0.85 (0.66–1.10)	
Female Gender *	1.29 (1.01–1.66) ***	1.39 (1.02–1.91) ***	0.92 (0.66–1.28)	
Migratory Background *	1.4 (0.94–2.09)	1.49 (0.98–2.26)	1.44 (0.94–2.20)	
Intermediate School Tier *	0.83 (0.38–1.85)	1.11 (0.49–2.47)	0.73 (0.44–1.18)	
Highest School Tier *	1.78 (0.78–4.08)	1.33 (0.64–2.78)	1.33 (0.83–2.11)	

OR: Odds ratio; CI: Confidence interval; * Reference categories are male gender, no migrant background, and lowest school tier. ** number of participants included in the regression model; *** *p* < 0.05.

**Table 5:** Correct answers on STI cures and vaccinations.

Question	Correct Response	n (%) Correct
HIV cure (n = 1131 *)	no reliable cure	946 (83.6%)
Hepatitis B cure (n = 1122 *)	no reliable cure	245 (21.8%)
Genital herpes cure (n = 1121 *)	no reliable cure	88 (7.9%)
HPV cure (n = 1126 *)	no reliable cure	25 (2.2%)
Chlamydia cure (n = 1125 *)	cure exists	212 (18.8%)
HIV vaccination (n = 1133 *)	no vaccination	716 (63.2%)
Hepatitis B vaccination (n = 1134 *)	vaccination exists	552 (48.7%)
Genital herpes vaccination (n = 1125 *)	no vaccination	200 (17.8%)
HPV vaccination (n = 1133 *)	vaccination exists	122 (10.8%)
Chlamydia vaccination (n = 1130 *)	no vaccination	113 (10.0%)

* number of responses included.

**Table 6:** Correct answers on STI cures and vaccinations by gender.

Question	Gender	Percentage Correct	*p* (from χ^2^)
HIV cure	male	82.5%	0.29
n = 1116 *	female	85.0%	
Hepatitis B cure	male	24.4%	0.03
n = 1108 *	female	19.0%	
Genital herpes cure	male	11.0%	<0.001
n = 1107 *	female	4.2%	
HPV cure	male	2.6%	0.41
n = 1113 *	female	1.7%	
Chlamydia cure	male	18.3%	0.76
n = 1112 *	female	19.1%	
HIV vaccination	male	66.8%	0.02
n = 1119 *	female	59.8%	
Hepatitis B vaccination	male	45.5%	0.02
n = 1120 *	female	52.4%	
Genital herpes vaccination	male	17.4%	0.70
n = 1111 *	female	18.3%	
HPV vaccination	male	12.9%	0.01
n = 1119 *	female	7.9%	
Chlamydia vaccination	male	8.8%	0.23
n = 1116 *	female	11.0%	

* number of responses included.

**Table 7:** Factors associated with correct responses on cure and vaccination questions.

**Variable**	**HIV** n = 1109 *	**Hepatitis B** n = 1101 *	**Genital Herpes** n = 1100 *	**HPV** n = 1106 *	**Chlamydia** n = 1105 *
	**OR (95% CI)**	**OR (95% CI)**	**OR (95% CI)**	**OR (95% CI)**	**OR (95% CI)**
Age (per year increase)	1.00 (0.82–1.22)	1.23 (0.94–1.61)	1.14 (0.90–1.45)	1.02 (0.86–1.22)	1.29 (1.08–1.54) **
Female Gender *	1.14 (0.82–1.58)	0.93 (0.71–1.22)	0.37 (0.22–0.61) **	0.91 (0.68–1.21)	1.13 (0.83–1.52)
Migratory Background *	0.73 (0.47–1.12)	0.82 (0.53–1.28)	1.22 (0.82–1.81)	1.50 (1.06–2.12) **	0.86 (0.65–1.14)
Intermediate School Tier *	3.14 (1.56–6.29) **	1.55 (1.02–2.34) **	1.89 (0.79–4.55)	0.98 (0.55–1.77)	1.62 (1.16–2.26) **
Highest School Tier *	2.32 (1.11–4.84) **	1.43 (0.88–2.32)	0.41 (0.13–1.29)	0.83 (0.57–1.22)	1.05 (0.60–1.83)
	**HIV** n = 1112 *	**Hepatitis B** n = 1113 *	**Genital Herpes** n = 1104 *	**HPV** n = 1112 *	**Chlamydia** n = 1109 *
	**OR (95% CI)**	**OR (95% CI)**	**OR (95% CI)**	**OR (95% CI)**	**OR (95% CI)**
Age (per year increase)	1.10 (0.98–1.25)	1.07 (0.89–1.27)	1.13 (0.86–1.49)	1.29 (0.93–1.79)	1.14 (0.88–1.47)
Female Gender *	0.70 (0.47–1.04)	1.30 (0.99–1.70)	1.09 (0.80–1.47)	0.60 (0.41–0.88) **	1.24 (0.94–1.64)
Migratory Background *	0.61 (0.43–0.88) **	0.82 (0.69–0.98)	0.75 (0.53–1.07)	0.74 (0.45–1.23)	0.71 (0.47–1.07)
Intermediate School Tier *	2.59 (1.71–3.94) **	1.31 (0.83–2.07)	1.61 (0.79–3.29)	1.77 (1.10–2.87) **	1.88 (1.34–2.63) **
Highest School Tier *	1.70 (1.01–2.86) **	1.14 (0.77–1.68)	1.12 (0.43–2.92)	0.65 (0.29–1.46)	1.12 (0.72–1.73)

OR: Odds ratio; CI: Confidence interval; * number of participants included in the regression model; ** *p* < 0.05.

## Supplementary Materials

The following are available online at www.mdpi.com/1660-4601/15/1/110/s1, Supplementary File 1: lack of awareness of STIs by gender, migrant background, and school type, Supplementary File 2: Correct answers on STI cures and vaccinations by migrant background, Supplementary File 3: Correct answers on STI cures and vaccinations by school type.
