# Supplementary material for: STI Knowledge in Berlin Adolescents
Source: Int J Environ Res Public Health. 2018 Jan 10;15(1):110. doi: 10.3390/ijerph15010110 (PMC5800209; doi:10.3390/ijerph15010110)
Supplement: Supplementary file 1 [file ijerph-15-00110-s001.zip › Supplementary File 3 - Schooltype and Knowledge.docx]

Supplementary File 3 - Correct answers on STI cures and vaccinations by school type.

|  | **HIV cure n=1131*** | **Hepatitis B cure n=1122*** | **Herpes cure n=1121*** | **HPV cure n=1126*** | **Chlamydia cure n=1125*** |
| --- | --- | --- | --- | --- | --- |
| **School Type** | n (%**) | n (%**) | n (%**) | n (%**) | n (%**) |
| lowest tier | 276 (74.4%) | 75 (20.4%) | 35 (9.6%) | 10 (2.7%) | 62 (16.8%) |
| intermediate tier | 347 (90.6%) | 98 (26.1%) | 41 (10.9%) | 9 (2.4%) | 86 (22.8%) |
| highest tier | 323 (85.7%) | 94 (24.9%) | 12 (3.2%) | 6 (1.6%) | 64 (16.9%) |
| p (from χ2) | <.001 | .16 | <.001 | .57 | .06 |
|  | **HIV vacc. n=1133*** | **Hepatitis B vacc.  n=1134*** | **Herpes vacc. n=1125*** | **HPV vacc. n=1133*** | **Chlamydia vacc. n=1130*** |
| **School Type** | n (%**) | n (%**) | n (%**) | n (%**) | n (%**) |
| lowest tier | 201 (54.2%) | 168 (45.3%) | 62 (16.8%) | 39 (10.6%) | 28 (7.6%) |
| intermediate tier | 279 (73%) | 200 (52.5%) | 74 (19.6%) | 58 (15.1%) | 52 (13.6%) |
| highest tier | 236 (62.1%) | 184 (48.2%) | 64 (16.9%) | 25 (6.6%) | 33 (8.8%) |
| p (from χ2) | <.001 | .14 | .53 | .001 | .01 |

*number of participants included in the analysis; **percentage of correct responses within subgroup
