# Supplementary material for: STI Knowledge in Berlin Adolescents
Source: Int J Environ Res Public Health. 2018 Jan 10;15(1):110. doi: 10.3390/ijerph15010110 (PMC5800209; doi:10.3390/ijerph15010110)
Supplement: Supplementary file 1 [file ijerph-15-00110-s001.zip › Supplementary File 2 - Migration and Knowledge.docx]

Supplementary File 2 - Correct answers on STI cures and vaccinations
by migrant background.

|  | **HIV  n=1121*** | **Hepatitis B  n=1112*** | **Herpes  n=1111*** | **HPV n=1116*** | **Chlamydia n=1115*** |
| --- | --- | --- | --- | --- | --- |
| **Migrant Background** | n (%**) | n (%**) | n (%**) | n (%**) | n (%**) |
| Both parents German-born | 463 (87.5%) | 135 (25.5%) | 39 (7.4%) | 4 (0.8%) | 112 (21.2%) |
| Both parents born abroad | 252 (74.3%) | 62 (18.7%) | 25 (7.5%) | 10 (3%) | 48 (14.3%) |
| Only mother born abroad | 108 (87.8%) | 34 (28.1%) | 15 (12.3%) | 7 (5.7%) | 25 (20.7%) |
| Only father born abroad | 114 (87.7%) | 32 (24.6%) | 9 (7.1%) | 3 (2.3%) | 26 (19.8%) |
| p (from χ2) | <.001 | .08 | .31 | .004 | .08 |
|  | **HIV  n=1123*** | **Hepatitis B  n=1124*** | **Herpes  n=1115*** | **HPV n=1123*** | **Chlamydia n=1120*** |
| **Migrant Background** | n (%**) | n (%**) | n (%**) | n (%**) | n (%**) |
| Both parents German-born | 370 (70.1%) | 276 (51.9%) | 115 (21.9%) | 63 (11.9%) | 61 (11.6%) |
| Both parents born abroad | 177 (51.6%) | 156 (45.9%) | 41 (12.1%) | 36 (10.5%) | 29 (8.5%) |
| Only mother born abroad | 84 (68.9%) | 52 (43%) | 21 (17.4%) | 10 (8.3%) | 13 (10.8%) |
| Only father born abroad | 81 (62.3%) | 64 (48.9%) | 19 (14.6%) | 13 (10%) | 10 (7.6%) |
| p (from χ2) | <.001 | .19 | .002 | .66 | .37 |

*number of participants included in the analysis; **percentage of correct responses within subgroup
