# Supplementary material for: STI Knowledge in Berlin Adolescents
Source: Int J Environ Res Public Health. 2018 Jan 10;15(1):110. doi: 10.3390/ijerph15010110 (PMC5800209; doi:10.3390/ijerph15010110)
Supplement: Supplementary file 1 [file ijerph-15-00110-s001.zip › Supplementary File 1 - lack of awareness by gender, migrant bg, school type.docx]

Supplementary File 1 – Complete lack of awareness of different STIs by gender, migrant background, and school type.

|  | **HIV** | **Hepatitis B** | **Herpes** | **Syphilis** | **HPV** | **Gonorrhea** | **Chlamydia** |
| --- | --- | --- | --- | --- | --- | --- | --- |
| **Variable** | n (%) | n (%) | n (%) | n (%) | n (%) | n (%) | n (%) |
| **gender**  female | 5 (0.9%) | 70 (13.2%) | 138 (26.1%) | 214 (40.3%) | 220 (41.6%) | 254 (48.0%) | 246 (46.2%) |
| male | 12 (2.0%) | 79 (13.4%) | 127 (21.6%) | 165 (28.2%) | 199 (33.9%) | 224 (38.4%) | 274 (46.6%) |
| n* | 1134 | 1122 | 1116 | 1117 | 1116 | 1112 | 1121 |
| p (from χ2) | .13 | .91 | .08 | <.001 | .01 | .001 | .88 |
| **migrant background**  both parents German-born | 3 (0.6%) | 52 (9.8%) | 79 (14.9%) | 139 (26.2%) | 156 (29.3%) | 181 (34.3%) | 212 (39.8%) |
| both parents born abroad | 12 (3.5%) | 55 (16.2%) | 121 (36.2%) | 149 (44.2%) | 165 (49.4%) | 174 (52.3%) | 184 (54.9%) |
| mother born abroad | 0 (0.0%) | 17 (13.6%) | 30 (24.4%) | 46 (37.7%) | 51 (42.1%) | 59 (48.0%) | 63 (50.4%) |
| father born abroad | 1 (0.8%) | 24 (18.3%) | 35 (26.5%) | 43 (32.6%) | 45 (34.1%) | 63 (47.7%) | 60 (45.5%) |
| n* | 1138 | 1126 | 1120 | 1121 | 1119 | 1116 | 1124 |
| p (from χ2) | .001 | .01 | <.001 | <.001 | <.001 | <.001 | <.001 |
| **school type**  lowest tier | 13 (3.4%) | 51 (13.6%) | 99 (26.5%) | 130 (35.3%) | 125 (33.7%) | 139 (38.1%) | 167 (45.1%) |
| intermediate tier | 1 (0.3%) | 41 (10.7%) | 63 (16.6%) | 109 (28.4%) | 116 (30.5%) | 159 (41.6%) | 150 (39.1%) |
| highest tier | 3 (0.8%) | 59 (15.6%) | 104 (27.5%) | 141 (37.2%) | 181 (47.9%) | 183 (48.4%) | 207 (54.5%) |
| n* | 1148 | 1136 | 1130 | 1131 | 1129 | 1125 | 1134 |
| p (from χ2) | .001 | .13 | <.001 | .03 | <.001 | .02 | <.001 |

*number of participants included in the analysis
